# Influence of High-Pressure Homogenization on the Physicochemical Properties and Betalain Pigments of Red Beetroot (*Beta vulgaris* L.) Juice

**DOI:** 10.3390/molecules28052018

**Published:** 2023-02-21

**Authors:** Bartosz Kruszewski, Ewa Domian, Małgorzata Nowacka

**Affiliations:** 1 Department of Food Technology and Assessment, Institute of Food Sciences, Warsaw University of Life Sciences—SGGW, 02-776 Warsaw, Poland; 2 Department of Food Engineering and Process Management, Institute of Food Sciences, Warsaw University of Life Sciences—SGGW, 02-776 Warsaw, Poland

**Keywords:** high-pressure homogenization, beetroot juice, betalains, betanin, HPLC-DAD, color, juice turbidity, PCA analysis

## Abstract

High-pressure homogenization (HPH) is considered an innovative and modern method of processing and preserving liquid and semi-liquid foods. The aim of this research was to examine the impact of HPH processing on the content of betalain pigments and physicochemical properties of beetroot juice. Combinations of the following HPH parameters were tested: the pressure used (50, 100, 140 MPa), the number of cycles (1 and 3) and the applied cooling or no cooling. The physicochemical analysis of the obtained beetroot juices was based on the determination of the extract, acidity, turbidity, viscosity and color values. Use of higher pressures and a greater number of cycles reduces the turbidity (NTU) of the juice. Moreover, in order to maintain the highest possible extract content and a slight color change of the beetroot juice, it was crucial to perform sample cooling after the HPH process. The quantitative and qualitative profiles of betalains have been also determined in the juices. In terms of the content of betacyanins and betaxanthins, the highest values were found in untreated juice at 75.3 mg and 24.8 mg per 100 mL, respectively. The high-pressure homogenization process resulted in a decrease in the content of betacyanins in the range of 8.5–20.2% and of betaxanthins in the range of 6.5–15.0%, depending on the parameters used. Studies have shown that that the number of cycles was irrelevant, but an increase in pressure from 50 MPa to 100 or 140 MPa had a negative effect on pigment content. Additionally, juice cooling significantly limits the degradation of betalains in beetroot juice.

## 1. Introduction

Red beetroots (*Beta vulgaris* L.) and juice made from them are becoming more and more valuable to consumers worldwide due to the growing number of scientific reports on the health benefits of their consumption [1,2]. Beetroot of many varieties and shapes is cultivated on all continents in the temperate climate zone. According to recent reports, Poland is the largest producer of red beets in the European Union (EU) with a 35% share in total production [3]. This vegetable is exported primarily to Slovakia (4.3 thousand tons, 24% share of export volume), Czech Republic (3.6 thousand tons, 20%) and Romania (2.3 thousand tons, 13%). Ukraine was also a significant buyer (1.8 thousand tons, 10%) [3]. Beetroot is eagerly consumed in Poland and Europe (about 8% of total EU vegetables volume consumed). Due to its good storability, it can be available fresh for almost the entire year.

Red beetroot is considered a health-promoting food due to the presence of nutritional and bioactive components such as vitamins, minerals, phenols, nitrates and betalains [4]. It contains vitamins such as C, A, E and K and is also abundant in vitamins from the B group [5]. Beetroot is not only a source of vitamins but also of minerals, which include manganese, magnesium, potassium, sodium, phosphorus, iron, zinc, copper, boron, silicon and selenium [5]. Thanks to its high fiber content, it has a beneficial effect on digestive processes [6]. Beetroot is among the top ten vegetables most abundant in antioxidant compounds [4,7]. The flesh and juice contain high amount of flavonoids, flavonols, otho-diphenols, condensed tannins and other substances classified to antioxidants [8]. It is also easily digestible and low in calories [5]. Red beetroot is a valuable raw material commonly used in the processing industry for the production of various types of dried, frozen, fermented and canned foods, as well as juices and their concentrates [9]. Most importantly, it is used in food industry in the form of juice concentrate as a coloring food and as a raw material for the extraction of the natural food additive betanin dye E162 [6].

The main source of betalains in nature is beetroot (*B. vulgaris* L.), especially its peel, but they are also found in some parts of amaranth and the fruits of *Opuntia* and *Hylocerasus* cactus, as well as in mushroom species such as *Amanita muscaria* [10]. The pigments are water-soluble and divided into two groups in terms of their molecular structure: red betacyanins and yellow betaxanthins. Their quantitative ratio determines the color and it depends on the plant variety [11]. The predominant pigment is betanin, which belongs to the betacyanins group. However, all betalains exhibit antioxidant properties, with proven lipid peroxidation preventive activity [9,12]. Scientific studies have shown that the stability of betalains is affected by pH, water activity, metal cations (such as iron, copper, tin, aluminum), oxygen concentration, light availability and the presence of endogenous enzymes and antioxidant compounds [6,9]. Processing parameters that need to be monitored because they affect betalain content in food include temperature and duration of heating, oxygen availability and pigment concentration [10,12].

High-pressure homogenization (HPH) together with high hydrostatic pressure (HHP) belongs to a group of innovative food processing and preservation methods based on application of high pressure [13,14]. Originally, HPH was only used to produce good quality emulsions and homogenize complex liquid products as a standardization step. It involves forcing liquid or semi-liquid products under pressure through a valve with a narrow gap of different geometries [13,15]. As a result of physical phenomena such as friction, collision, cavitation and turbulence, a mechanical reduction of particles and disruption of microbial cells occurs [16,17]. A side effect is a temperature increase on the valve and the product, dependent on height of the pressure drop within the valve. Therefore, in order to reduce the effect of temperature on bioactive compounds in the product, cooling of the valve or product after it exits the device is used [18,19].

Previous studies show a different effect of HPH on biologically active compounds in food, in relationship to the pressure applied, the temperature of the product at the input, and the number of product passes through the homogenizer [20,21]. Depending on the parameters and type of the pigments, experiments showed no effect or a decrease in content at level of 10–30%. An increase in pigments concentration up to 10% of the original value was also reported in some cases [18,19]. However, there is no information in the literature about the effect of the HPH method on the concentration of betalains. Therefore, the main objective of the study was to evaluate the effect of HPH betalain pigments of beetroot juice. Changes in basic physicochemical parameters (total soluble solids, pH, titratable acidity, direct turbidity, serum cloudiness, color and viscosity) were also investigated.

## 2. Results and Discussion

### 2.1. Temperature Changes during the HPH Processing

High-pressure homogenization treatment increased the temperature of the juices. The temperature increase was proportional to the increase of pressure used during processing. For homogenized samples at 50, 100 and 140 MPa, the juice temperature measured at the outlet of the device after cycle increased by a maximum of 7.4, 14.4 and 21.1 °C, respectively (Table 1). The influence of applied pressure is very evident. This is due to the physical phenomena occurring in the homogenizing valve such as shear, cavitation, turbulence and impacts with surfaces, that become more intense as the pressure increases. According to Dumay et al. [15] a total increase of temperature of various products falls within the range of 17–21 °C per 100 MPa. It comprises (1) fluid temperature increase with the homogenization pressure by 2–3 °C per 100 MPa, due to the heat of compression generated during the pressure build-up in the pressure intensifier; (2) linear increase with the homogenization pressure by 14–18 °C per 100 MPa, due to shear effects and partial conversion of mechanical energy into heat. Most of the pressure is dissipated as heat, and only a small part is used for mechanical disintegration of particles. In the present study there was obtained a temperature rise lower than the given relation, but it should be noted that the intensity of physical phenomena during the process is affected by the architecture of the valve, valve construction material characteristics and the composition of the homogenized product. Our homogenizer used for experiments had a sharp-angle type valve, and was made of abrasion- and corrosion-resistant ceramic and Duplex stainless steel.

The use of relatively low inlet temperatures of the product in the HPH processing is one of the methods to preserve thermolabile bioactive components. However, it is not always advisable to use a low inlet temperature because less deactivation of microorganisms and enzymes is obtained [22,23]. A second method is cooling after the process with a heat exchanger or cooling the valve [19,24].

HPH processing at 140 MPa without cooling after one cycle caused a +21.2 °C rise in juice temperature. However, after three cycles, it resulted in a relatively small temperature rise (change +15.8 °C) compared to the temperature recorded after one cycle under the same conditions (Table 1). This was surprisingly less than expected. The temperature of the product was not increasing by 21 °C on each cycle, because the beetroot juice was passively cooled from the temporary collection container awaiting the next entry into the homogenizer. In addition, the juice was also passively cooled on the components of the device, as only the valve itself had an elevated temperature. The ambient temperature during the processing was about 19 °C.

### 2.2. Total Soluble Solids (TSS), pH and Titratable Acidity (TA)

According to the results (Table 2), the high-pressure homogenization has not affected the TSS content of the beetroot juice when cooling was applied. Significant changes in the reduction of TSS occurred (−0.4 °Brix) when the same treatment was applied but without cooling. Apparently, the presence of heat reduced the extract. The literature states that under the influence of elevated temperature residual protein precipitation and depletion of reducing sugars takes place [25,26]. No effect of pressure parameters and number of cycles was observed on the TSS content of the juice. In a previous study on blackcurrant juice [18], as well as in the present research, there was no impact of the number of cycles on extract content, but the effect of pressure was significant. However, the reduction in TSS occurred only as a result of the pressure change to the level of 220 MPa. Other researchers have found no change in TSS in juices under high-pressure homogenization [17,21] or have pointed to a direct impact of pressure and number of cycles [26].

pH and TA are very important parameters of beverages from a safety point of view and have a direct impact on the choice of preservation methods and storage conditions. The results show no change in the TA of the juice, while changes were observed in pH (Table 2). The pH value slightly but statistically significantly increased by an average of 0.13 in all homogenized samples regardless of whether cooling was used. This could have been due to the extraction of alkaline-forming compounds from residual particles. Our results are in contrast with those reported by Velázquez-Estrada et al. [24] who observed no changes in pH and significant decrease of TA in HPH-processed orange juice. The authors pointed to a greater influence of inlet temperature than pressure alone on pH and TA characteristics. However, it should be noted that beetroot juice, in contrast to orange juice, has a different composition of sugars, organic acids and bioactive compounds, so the effect of HPH may have been different.

### 2.3. Direct Turbidity and Serum Cloudiness

**Figure 1:**
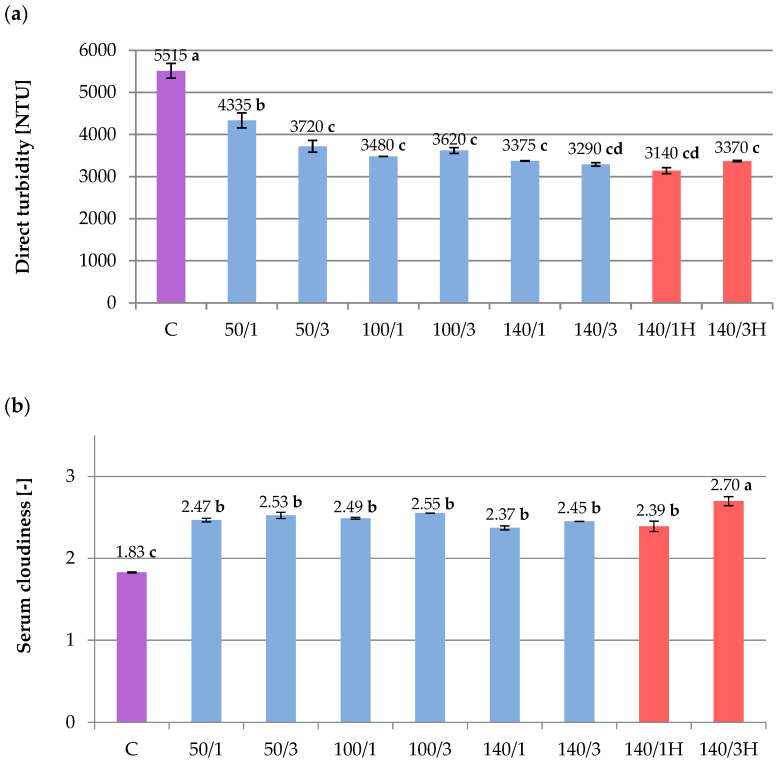
Influence of HPH processing on beetroot juice parameters: (**a**) Direct turbidity (NTU). (**b**) Serum cloudiness (absorbance at 660 nm). C—control; 50/1, 50/3, etc.,—pressure/number of cycles; H—samples without cooling. The values with different letters are significantly different (*p* < 0.05).

Measurement of turbidity (NTU) indicated that very cloudy beetroot juice was obtained (Figure 1a). Turbidity is usually formed by pectin, fats, cellulose compounds, proteins and their complexes with various substances, as well as other compounds [27]. Based on the results of the study, there is a significant effect of the homogenization process on the turbidity of all juice samples. The number of cycles was relevant only at 50 MPa. Increasing the pressure from 50 MPa to 100 or 140 MPa resulted in an even greater reduction in turbidity, although no statistical difference was observed between 100 and 140 MPa. The best effect of reducing NTU among all juice samples was achieved in the variant with a pressure of 140 MPa, especially the three cycles with cooling, and the one cycle without cooling. The HPH process mechanically reduces the size of particles suspended in the juice matrix [27], which allows more light to pass through without reflection. Additionally, in HPH samples without cooling, the elevated temperature may have led to particle precipitation as suggested by the TSS values (Table 2). In an earlier publication, the impact of pressure parameters and number of cycles on the direct turbidity of blackcurrant juice was also observed, but was more intense [18].

Serum cloudiness of the raw beetroot juice, presented in the Figure 1b, was high (1.83) compared to the values obtained in other juices reported in the literature. Velázquez-Estrada et al. [24] obtained serum cloudiness at the level of 0.46 in orange juice, while Silva et al. [27] measured it at an average of 0.26 in pineapple pulp. These values suggest that the beetroot juice from our study had a more colloidal matrix. Homogenization resulted in an increase in serum turbidity in all variants (Figure 1b). The sample with the most severe parameters (140 MPa, three cycles, no cooling) in the experiment had the highest value of about 1.5 times the original value. In view of the above, the matrix of the juice has become even more colloidal, which potentially stabilized the juice and delayed sedimentation during further storage. Some researchers link the increase in serum turbidity to a decrease in particle size [20,24].

### 2.4. Influence of HPH on Juice Color and Viscosity

HPH treatment had a direct effect on the color of the red beetroot juice (Table 3). Samples processed at 50, 100 and 140 MPa but with only one cycle had a similar color, slightly altered from the raw juice (ΔE* in range 0.13–0.17). Greater, statistically significant changes in color were noted when the juice was homogenized three times (ΔE* in range 0.29–0.43). Samples that were not cooled after homogenization had the highest ΔE* values, 0.69 and 0.94 for 140 MPa/1 cycle and 140 MPa/3 cycles, respectively. The only color parameter that changed in all samples was the a*, which was decreasing as a result of more severe HPH parameters. The reason is the degradation of the juice’s betalain pigments, particularly betacyanins, as discussed in Section 2.5. Scientific reports describe various influences of HPH on product color parameters. In processed blackcurrant juice, both L* and b* values increased, while the a* value decreased, resulting in obtained ΔE* value at 3.33 [18]. HPH treatment of strawberry-based smoothie impacted L* and a* value positively [28]. The changes were explained by modifications in particle size and shape, their aggregation, oxidative reactions and caramelization of fruit sugars. However, all of these studies were conducted on different HPH equipment, and in addition, the products had different formulation.

The viscosity of a liquid is the internal friction that occurs during its flow. It can be described also as a resistance of a liquid to a change in shape, or movement of neighboring portions relative to one another. Knowledge of the physical properties, including rheological parameters such as viscosity of liquid food products is very important during the design of processes and industrial equipment, as well as at the stage of product development. Based on the study results, it can be stated that the HPH process reduced the viscosity of the beetroot juice. The cooling intervention during HPH did not significantly affect the viscosity values (Table 3). The process of HPH breaks up solid particles in suspensions, and consequently, the viscosity of the fluid decreases or increases depending on the type of matrix and the particles contained in it, as well as their size. Szczepańska et al. [29] obtained a significant decrease in the viscosity of apple juice after applying HPH; they observed the lowest viscosity at 200 MPa. According to the observations, this could be related to more significant changes in particle size distribution (greater reduction in particle size). In contrast, other researchers reported an increase of apparent viscosity in mango juice after HPH processing [30]. However, they suggested that the increase could have been partially due to an increase in the solubility of high molecular weight carbohydrates such as starch and pectin. Furthermore, inactivation of pectin-degrading enzymes by HPH prevents pectin depolymerization, resulting in higher serum viscosity and higher juice consistency [30].

### 2.5. Qualitative and Quantitative Determinations of Betalains

**Figure A1:**
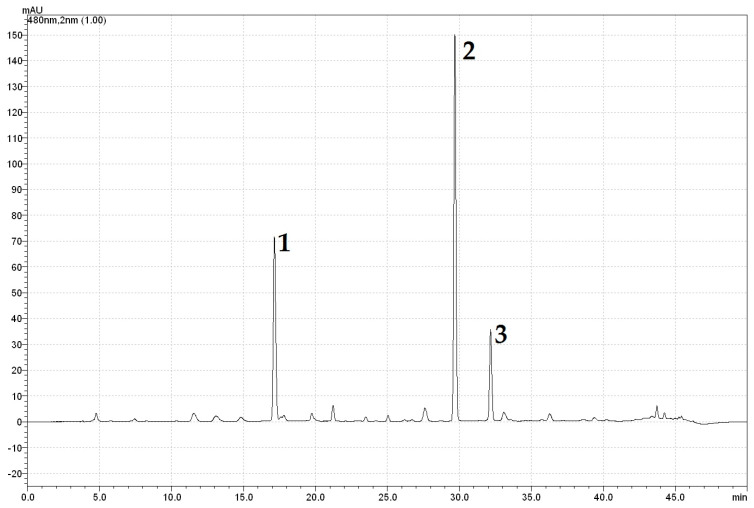
HPLC chromatogram of betalains at a wavelength of 480 nm: 1—vulgaxanthins I and II, 2—betanin, 3—isobetanin.

**Figure A2:**
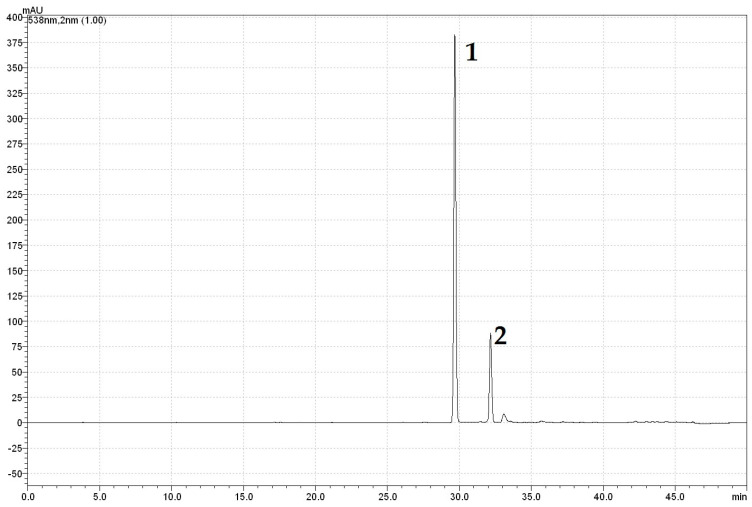
HPLC chromatogram of betalains at a wavelength of 538 nm: 1—betanin, 2—isobetanin.

High-performance liquid chromatography with diode-array detection (HPLC-DAD) analysis allowed identification of the betacyanins compounds in beetroot juice samples: betanin, isobetanin (Figure A1) and the betaxanthins vulgaxanthin I and II (Figure A2). The conditions of the chromatographic analysis allowed clear separation of the individual components. Due to the lack of commercially available quantitative standards, the concentration of betalain pigments was determined spectrophotometrically. Changes in betalain pigments determined both by the chromatographic and spectrophotometric methods after the HPH process are shown in Table 4.

Losses of both groups of betacyanins were observed in juice samples. Among the betacyanins, the betanin was the most resistant pigment to the process conditions. However, the losses of betanin, defined as the change in peak area, were 12.7%, 15.2% and 16.2% for pressures of 50, 100 and 140 MPa with cooling, respectively. For isobetanin, the losses were 14.2%, 18.2% and 19.8% for 50, 100 and 140 MPa with cooling, respectively. There was statistically significant impact of pressure, but no effect of the number of cycles on the betanin or isobetanin peak area. Increased heat dosage in samples without cooling resulted in even greater betanin and isobetanin degradation. Moreover, in this type of treatment, three cycles resulted in a statistically significantly higher degradation of isobetanin compared to one cycle (Table 4). Quantification of total betacyanins confirms chromatographic studies. The linear correlation analysis performed between these two determinations resulted in a Pearson’s correlation coefficient of r = 0.981 and determination coefficient r^2^ = 0.962. The content of betacyanins dropped from 75.3 mg/100 mL to the minimum value of 65.3 mg/100 mL (about 13%) in HPH cooled juice samples. Additionally, an increased degrading effect of lack of cooling during HPH process on betacyanins was observed (total 20% reduction).

The chromatographic determination of vulgaxanthin I and II also showed an effect of HPH treatment. However, losses were less than recorded for betanin and isobetanin (Table 4). There was an influence of homogenization pressure but no effect of the number of cycles on the peak area of vulgaxanthins. The smallest degradation was caused by a pressure of 50 MPa, followed by pressures of 100 and 140 MPa (these two caused similar decreases). HPH samples without cooling showed even more progressive vulgaxanthin degradation. The linear correlation analysis performed between chromatographic and spectrophotometric determinations of betaxanthin pigments resulted in a Pearson’s correlation coefficient of r = 0.960 and determination coefficient r^2^ = 0.921. The content of betaxanthins dropped from 24.8 mg/100 mL to the minimum value of 22.1 mg/100 mL (about 11%) in HPH cooled juice samples. The not-cooled samples had minimally lower concentrations of these substances (total 15% reduction).

According to the literature, betalain pigments are more stable to negative processing and storage conditions such as temperature, pH, light and presence of oxygen than anthocyanins commonly present in food [31,32]. This is confirmed after comparing to the results of anthocyanin concentrations in blackcurrant juices previously processed under similar HPH parameters [18]. Both individual anthocyanin monomers and their total content recorded higher levels of degradation than content of betalains in present study. Furthermore, it was observed that betaxanthins were more resistant to HPH processing conditions than betacyanins. We presume that the degradation of betalains in the beetroot juice during HPH treatment was partially due to oxidation reactions with the oxygen present in the juice, and partially due to exposure of these compounds to endogenous enzymes. To some extent these reactions were limited by reducing substances such as flavonoids, flavonols, phenolic acids and other antioxidants, which are abundant in red beetroot. In addition, in the HPH samples without cooling, there is an additional degradation mechanism in the form of the thermal induction of hydrolysis of the aldimine bond of betanin and isobetanin with production of the betalamic acid and cyclo-Dopa 5-O-β-glucoside [31]. This is favored by a pH > 6, which characterized the samples. According to Skalicky et al. [33], the effect of high temperature can also cause the loss of conjugated sugar, which leads to the formation of labile aglycones with a different λmax. Another mechanism of thermal degradation of betanin and isobetanin involves decarboxylation and dehydrogenation. However, the loss of one carboxyl group did not affect the betanidin chromophore, and the resulting molecule is even more stable [33,34]. Other researchers confirm the effect of elevated temperatures on betalains’ degradation [35,36].

### 2.6. Comprehensive Overwiew of All Samples—PCA Analysis Results

**Figure 2:**
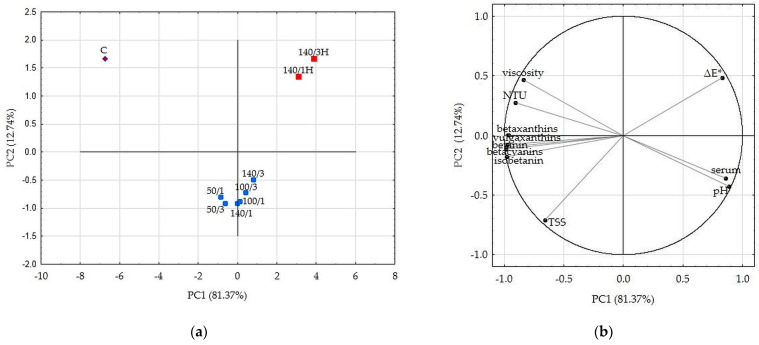
PCA results: (**a**) Score plot, PC1 versus PC2 of all samples. (**b**) Score plot, PC1 versus PC2 of data from determinations used as variables. C—control sample; HPH samples marked as blue squares are labeled as level of pressure/number of cycles; HPH samples marked as red squares with H are samples without cooling.

Principal component analysis (PCA) figures, based on the first two principal components which explained 94.11% of the total variance, demonstrate grouping of the beetroot juice samples according to parameters of HPH treatment (Figure 2a,b).

Based on the PCA figures, the pressure parameters of 50, 100 and 140 MPa in combination with one or three cycles, when the samples were cooled, have formed a single group on the created plane. Samples of individual HPH juices differed in their composition and properties, as the group they formed is not so compact. Figure 2a shows that these samples are located far from the control sample, which means that each combination of HPH processing parameters significantly affected the characteristics of the juice. A separate group was formed by samples after HPH treatment but without cooling applied. These samples had the lowest content of betalain compounds and the highest difference in color compared to the raw juice, but some similar values of pH, NTU, viscosity and serum cloudiness compared to HPH samples with applied cooling. Based on PCA analysis, the pressure of 140 MPa and one or three cycles without cooling are not recommended for use.

## 3. Materials and Methods

### 3.1. Juice Preparation and HPH Treatment

The material for the study was the juice from red beetroots of the “Czerwona Kula” variety, harvested on a farm in the Mazovia Province, Poland. This variety was chosen for the study because it is rich in betalain pigments and dedicated to industrial processing. The raw material was washed, allowed to dry and then weighed. The juice was pressed using a RAVEN EWW002 slow-speed juicer (Poland) and filtered on a sterile 17-strand gauze. The process yield was 49% (v/m). The obtained juice was poured into a collecting vessel, then separated by volume into a control sample and samples for processing.

**Figure 3:**
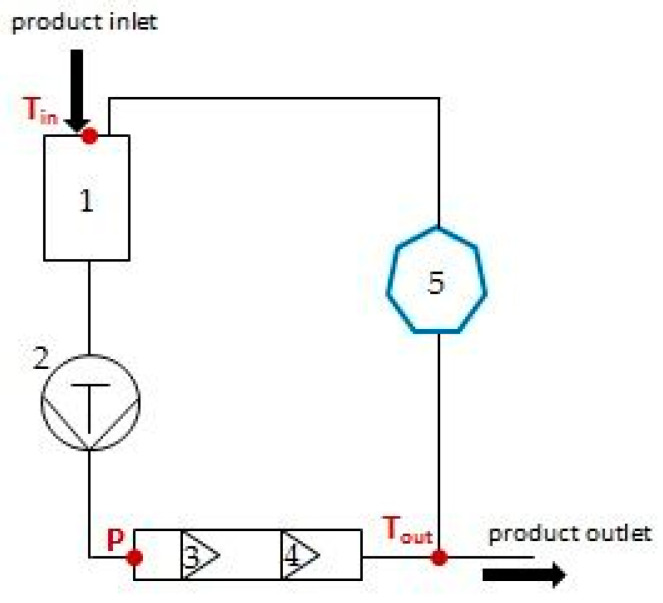
Schematic diagram of the high-pressure homogenizer used in the study. 1—feeding hopper, 2—piston pump, 3—first stage valve, 4—second stage valve, 5—intermediate container with cooling capability; T_in_—inlet temperature measured at the feeding hopper, P—summary pressure of first and second stage valves measured by digital manometer, T_out_—outlet temperature measured after leaving the head with the valves.

The high-pressure homogenization (HPH) process was carried out on a PANDA 2K NS1001L manufactured by GEA NIRO SOAVI (Parma, Italy). Only the first stage valve was used by setting pressures of 50, 100 and 140 MPa, at flow rate of 160 mL/min. According to the technical specification, the homogenizer has a sharp-angle type valve. Prior to the processing, the device was cleaned with 70% ethanol. At each pressure parameter, beetroot juice at an inlet temperature of 20.5 ± 0.5 °C was passed through the homogenization valve one and three times (1 and 3 cycles). All juice samples were cooled immediately in a container with an ice water bath after each cycle to reduce heat influence and adjust temperature to 20.5 ± 0.5 °C. In addition, for a pressure of 140 MPa, an additional series of HPH processing was carried out (1 and 3 cycles) but without cooling after each cycle. The temperature of all juice samples at the outlet of the device was monitored (Figure 3). The experiment with all HPH parameters variants was performed in two independent replicates.

### 3.2. Analysis of Total Soluble Solids, pH and Titratable Acidity

The extract content expressed as total soluble solids (TSS) was determined by placing few drops of juice on the measuring prism of the Refracto 30PX refractometer from Mettler-Toledo (Switzerland). The result was read at 20 °C directly from the device in °Brix. The measurement was performed in triplicate for each sample.

Both pH and titratable acidity (TA) of beetroot juice samples were analyzed in triplicate using an automated titrator TitroLine^®^ 5000 (SI Analytics^®^, Mainz, Germany). Before analyses, the titrator was calibrated with buffer solutions, and temperature of juice samples was adjusted to 23 °C. Titratable acidity was determined by titrating juice to pH 8.1 using 0.1 M sodium hydroxide. Results are expressed as g of citric acid per 100 mL of juice.

### 3.3. Direct Turbidity and Serum Cloudiness

The direct turbidity of the juice expressed in nephelometric turbidity units (NTU) was tested using a 2100 Q turbidimeter from HACH Lange GmbH (Berlin, Germany) based on the instrument’s instructions, at a range of 0–2000 NTU. Before analyses, the turbidimeter was calibrated against standards, and temperature of juice samples was adjusted to 23 °C. The analysis was made by placing into device a glass cuvette filled with diluted juice.

The adopted method of Wang et al. [21] was used to measure turbidity of the juice serum. Briefly, 6 mL of juice was placed in 15 mL tubes and centrifuged (20 °C, 10 min, 4200× *g*) on an MPW–352R device (MPW Med. Instruments, Warsaw, Poland). The supernatant was transferred into optical glass cuvettes and its absorbance was analyzed using a Shimadzu UV-1650PC spectrophotometer (Shimadzu Corp., Kyoto, Japan) at a wavelength of 660 nm. The absorbance result was directly related to the serum cloudiness.

Both measurements were carried out in triplicate.

### 3.4. Viscosity Measurement

Viscosity was measured using a Brookfield DV-II viscometer (AMETEK Brookfield, Middleborough, MA, USA) with adapter for a low viscosity samples and spindle No. 2. The result at a speed of 60 rpm was read from the display of the device in the mPa s unit. Measurements were carried out according to the device’s instructions in triplicate for each juice variant.

### 3.5. Color Parameters

Instrumental measurement of the beetroot juice’s color parameters was performed in the CIE L*a*b* system (L*—lightness; a*—red to green; b*—yellow to blue) using a Konica Minolta CM-3600d colorimeter (Osaka, Japan) [18]. Determination in fivefold repetition for each sample was made in transmission mode, with the following settings: an illuminant D65, an observation angle of 10°, using a glass cuvette with a layer thickness of 2 mm. The total color difference ΔE* between untreated and HPH-processed juice was calculated by the application of Equation (1).

(1) $${\Delta E}^{*}=\sqrt{{\left({\Delta L}^{*}\right)}^{2}+{\left({\Delta a}^{*}\right)}^{2}+{\left({\Delta b}^{*}\right)}^{2}}$$

### 3.6. Chromatographic Determination of Betalains

The qualitative determination of betalain compounds was carried out using a high-performance liquid chromatography coupled with diode array detector (HPLC-DAD) based on the methodology of Kujala et al. [37]. For this purpose, the beetroot juice was diluted with distilled water so that the absorbance values of the individual compounds will not be supersaturated in the DAD detector at 480 and 538 nm. The juice was filtered through an Alfatec hydrophilic PTFE syringe filter. The first four drops were discarded. Then, 1 mL of the diluted juice was taken into chromatography vials and capped.

Analysis of betalain compounds was carried out in a Shimadzu Modular HPLC (Shimadzu Corp., Japan) equipped with an LC-10ATvp pump, SPD-M20A DAD detector, CTD-10AsVp column thermostat and DGU-20A5R degasser. A Luna C18(2) 250 × 4.6 mm column from Phenomenex (Torrance, CA, USA) was used, with a pre-column mounted with the same characteristics. Two solvents were used: acetonitrile (A) and formic acid/water (0.4: 99.6, *v*/*v*) (B). The elution profile was 0–5 min, 100% B; 5–35 min, 0–13% A in B; 35–40 min, 13–27% A in B; 40–50 min, 100% B. The injection volume was 20 µL, the column temperature was set at 35 °C and the flow rate was 1.0 mL/min. The time of single analysis was 50 min.

Betanin and isobetanin (betacyanins representatives) were identified using a red beet extract standard (Sigma-Aldrich, Schnelldorf, Germany), and beetroot juice reconstituted from concentrate at a characteristic wavelength of 538 nm. Betaxanthins in the form of the sum of vulgaxanthins I and II were identified by comparing the chromatograms obtained with those published in the literature [37], taking into account the 480 nm wavelength. Three independent replicates were performed for each sample.

### 3.7. Spectrophotometric Quantification of Betalains

The content of betalain pigments was determined spectrophotometrically using a differential method according to Stintzing et al. [38]. With this method, pigments such as betacyanins (red-violet) and betaxanthins (yellow) can be determined simultaneously. All juice samples were diluted with the previously prepared McIlvaine buffer (citrate-phosphate buffer, pH adjusted to 6.5) in such a proportion that the absorbance value at 538 nm was in the range of 0.4–0.8. The blank sample was McIlvaine buffer alone. Absorbance was measured at three wavelengths: 476, 538 and 600 nm. All determinations were made in triplicate. The absorbance value of betacyanins (BC), taking into account light absorption due to the presence of various impurities in the matrix, was calculated according to Equation (2).

(2) $$A_{BC}=1.095\times \left(A_{538}-A_{600}\right)$$

The content of betacyanins, expressed in mg of betanin in 100 mL of juice, was calculated according to Equation (3).

(3) $$C_{\mathrm{BC}}=\frac{A_{\mathrm{BC}}\cdot \mathrm{DF}\cdot \mathrm{MW}\cdot 100}{\varepsilon \cdot L}$$

where DF—dilution factor; MW—molecular mass of betanin, 550 g/mol; ε—extinction coefficient for betanin, 60,000 L/mol*cm; L—thickness of the layer of the measured solution, 1 cm.

The absorbance value for betaxanthins (BX) at 476 nm taking into account the light absorbance due to the presence of impurities and betacyanins was calculated based on Equation (4).

(4) $$A_{\mathrm{BX}}={\;A}_{476}-A_{538}+0.677\cdot A_{\mathrm{BC}}$$

The content of betaxanthins, expressed in mg of vulgaxanthin in 100 mL of juice, was calculated using Equation (5).

(5) $$C_{\mathrm{BX}}=\frac{A_{\mathrm{BX}}\cdot \mathrm{DF}\cdot \mathrm{MW}\cdot 100}{\varepsilon \cdot L}$$

where DF—dilution factor; MW—molecular mass of vulgaxanthin, 390 g/mol; ε—extinction coefficient for vulgaxanthin, 48,000 L/mol*cm; L—thickness of the layer of the measured solution, 1 cm.

### 3.8. Statistics

All data are presented as a mean with standard deviation. Statistical analyses were conducted using Statistica 13.3 (TIBCO Software Inc., Palo Alto, CA, USA). The effect of HPH treatment on the physicochemical properties and betalain content of beetroot juice was analyzed using ANOVA analysis of variance. Any differences between the obtained values of the different juice variants were compared using the Tukey HSD test (α = 95%). Pearson’s correlation coefficients and determination coefficients between qualitative and quantitative data of betalains analyses were determined. The gathered data from the study were used in the principal component analysis (PCA) in order to comprehensively show the changes in physicochemical and betalain pigment profiles in the beetroot juice samples processed with different parameters using the HPH method. The gathered data (except titratable acidity values) were qualified for PCA analysis based on a correlation score with the first or second principal component of at least 0.6 [39].

## 4. Conclusions

In general, high-pressure homogenization (HPH) has a great potential to preserve the bioactive and physicochemical qualities of beetroot juice, but adequate parameters should be considered depending on the desired final characteristics of the juice. In our experiment, in terms of balance between good physicochemical qualities and betalains quantities in the juice, the best HPH treatment parameters were 100 and 140 MPa for one cycle with cooling. As the study proved, cooling of product during or after the HPH process is essential to reduce the level of loss in betalain pigments and to prevent the deterioration of the juice quality because of heating. As also shown, some physicochemical parameters and bioactive compounds are affected only by applied pressure, or the number of cycles, but at a certain pressure. There was a significant effect of HPH treatment on the juice turbidity (NTU), serum cloudiness, viscosity and pH.

The most resistant to the application of high pressure during homogenization are the vulgaxanthins I and II, as well as the entire group of betaxanthins. Among the betacyanins, it was betanin that had the lowest degradation rate. This is important, positive information for food and dietary supplement manufacturers because betanin makes up the majority of betalain pigments and is used as a food colorant.

## Figures and Tables

**Table 1:** Temperature changes during HPH processing, measured before cooling.

Pressure	Cooling after Each Cycle	Inlet Temperature [°C]	Maximum Temperature Monitored Directly after the Valve Head [°C]
50 MPa/1 and 3 cycles	yes	20.7 ± 0.5	28.1 ± 1.1
100 MPa/1 and 3 cycles	yes	20.6 ± 0.5	35.0 ± 1.2
140 MPa/1 and 3 cycles	yes	20.5 ± 0.5	41.6 ± 1.0
140 MPa/1 cycle H	no	20.6 ± 0.5	41.8 ± 1.2
140 MPa/3 cycles H	no	20.7 ± 0.5	57.6 ± 2.0

H—samples without cooling; temperature measured at the end of processing.

**Table 2:** The physicochemical characteristics: total soluble solids (TSS), pH and titratable acidity (TA) of beetroot juice samples treated with HPH.

Sample	pH	TA (g of Citric Acid/100 mL)	TSS (°Brix)
C	6.01 ± 0.02 ^a^	0.12 ± 0.01 ^a^	9.3 ± 0.1 ^a^
50 MPa/1 cycle	6.14 ± 0.01 ^b^	0.11 ± 0.01 ^a^	9.3 ± 0.1 ^a^
50 MPa/3 cycles	6.13 ± 0.01 ^b^	0.11 ± 0.01 ^a^	9.3 ± 0.1 ^a^
100 MPa/1 cycle	6.13 ± 0.01 ^b^	0.11 ± 0.01 ^a^	9.3 ± 0.1 ^a^
100 MPa/3 cycles	6.15 ± 0.00 ^b^	0.11 ± 0.01 ^a^	9.3 ± 0.1 ^a^
140 MPa/1 cycle	6.14 ± 0.00 ^b^	0.11 ± 0.01 ^a^	9.3 ± 0.1 ^a^
140 MPa/3 cycles	6.14 ± 0.00 ^b^	0.11 ± 0.01 ^a^	9.3 ± 0.1 ^a^
140 MPa/1 cycle H	6.16 ± 0.01 ^b^	0.11 ± 0.01 ^a^	8.9 ± 0.1 ^b^
140 MPa/3 cycles H	6.14 ± 0.00 ^b^	0.11 ± 0.01 ^a^	8.9 ± 0.1 ^b^

C—control; H—samples without cooling. The values in the same column with different letters are significantly different (*p* < 0.05).

**Table 3:** Influence of HPH processing on beetroot juice color and viscosity.

Sample	ΔE*	Viscosity (mPa s)
C	-	5.51 ± 0.02 ^b^
50 MPa/1 cycle	0.13 ± 0.04 ^a^	3.76 ± 0.04 ^a^
50 MPa/3 cycles	0.29 ± 0.01 ^b^	3.76 ± 0.05 ^a^
100 MPa/1 cycle	0.13 ± 0.04 ^a^	4.01 ± 0.05 ^a^
100 MPa/3 cycles	0.38 ± 0.02 ^c^	4.01 ± 0.07 ^a^
140 MPa/1 cycle	0.17 ± 0.03 ^a^	3.82 ± 0.06 ^a^
140 MPa/3 cycles	0.43 ± 0.03 ^c^	3.95 ± 0.04 ^a^
140 MPa/1 cycle H	0.69 ± 0.04 ^d^	3.76 ± 0.08 ^a^
140 MPa/3 cycles H	0.94 ± 0.06 ^e^	3.82 ± 0.05 ^a^

C—control; H—samples without cooling; ΔE*—total color difference between control and treated samples. The values in the same column with different letters are significantly different (*p* < 0.05).

**Table 4:** Betalain stability in beetroot juice samples treated with HPH.

Samples	Qualitative (% Relative Peak Area)			Quantitative (mg/100 mL)	
	Betanin	Isobetanin	Vulgaxanthin I and II	Betacyanins	Betaxanthins
Control—raw juice	100	100	100	75.3 ± 2.8 ^c^	24.8 ± 0.6 ^c^
Homogenization:					
50 MPa/1 cycle	87.2 ± 0.3 ^c^	85.5 ± 0.2 ^d^	92.6 ± 0.1 ^c^	68.9 ± 1.5 ^bc^	22.5 ± 0.6 ^ab^
50 MPa/3 cycles	87.5 ± 0.8 ^c^	86.2 ± 0.9 ^d^	93.0 ± 1.2 ^c^	67.1 ± 1.4 ^b^	23.2 ± 0.3 ^b^
100 MPa/1 cycle	85.3 ± 1.0 ^b^	82.0 ± 1.1 ^c^	88.9 ± 1.8 ^b^	66.6 ± 1.3 ^ab^	21.9 ± 0.1 ^ab^
100 MPa/3 cycles	84.4 ± 0.6 ^b^	81.6 ± 0.4 ^c^	89.6 ± 0.6 ^b^	66.4 ± 0.2 ^ab^	22.3 ± 0.1 ^ab^
140 MPa/1 cycle	83.7 ± 1.2 ^b^	81.4 ± 1.2 ^c^	89.1 ± 1.0 ^b^	67.3 ± 0.3 ^b^	22.8 ± 0.3 ^ab^
140 MPa/3 cycles	83.9 ± 0.2 ^b^	79.1 ± 0.3 ^c^	87.2 ± 0.4 ^b^	65.3 ± 1.3 ^ab^	22.1 ± 0.2 ^ab^
140 MPa/1 cycle H	75.4 ± 0.7 ^a^	70.9 ± 0.8 ^b^	83.8 ± 1.3 ^a^	62.6 ± 2.2 ^ab^	21.1 ± 0.3 ^a^
140 MPa/3 cycles H	74.4 ± 1.1 ^a^	66.0 ± 1.2 ^a^	83.7 ± 1.3 ^a^	60.1 ± 2.6 ^a^	21.2 ± 0.5 ^a^

The values in the same column with different letters are significantly different (*p* < 0.05). H—samples without cooling.

## Data Availability

All data created and analyzed during the experiments are presented in this study.
